# “Taking care of your pregnancy”: a mixed-methods study of group antenatal care in Kakamega County, Kenya

**DOI:** 10.1186/s12913-022-08200-1

**Published:** 2022-07-29

**Authors:** Aleefia Somji, Kate Ramsey, Sean Dryer, Fredrick Makokha, Constance Ambasa, Brittany Aryeh, Kathleen Booth, Serge Xueref, Seneca Moore, Ralpher Mwenesi, Shafia Rashid

**Affiliations:** 1 Management Sciences for Health (MSH), Alexandria, USA; 2 Scope, Brooklyn, USA; 3 Globally Minded Foundation, Burgas, Bulgaria; 4 Ministry of Health, Kakamega, Kenya; 5 Independent Consultant, Kakamega, Kenya; 6 Partners in Health (PIH), Boston, USA; 7 Columbia University Mailman School of Public Health, New York, USA; 8 MSH, Lyon, France; 9 Independent Consultant, Nice, France; 10 MSH, New York, USA

**Keywords:** Kenya, Pregnancy clubs, Quality of care, Experience of care, Empowerment, Social support, Group antenatal care

## Abstract

**Background:**

Traditional antenatal care (ANC) models often do not meet women’s needs for information, counseling, and support, resulting in gaps in quality and coverage. Group ANC (GANC) provides an alternative, person-centered approach where pregnant women of similar gestational age meet with the same health provider for facilitated discussion. F studies show associations between GANC and various outcomes.

**Methods:**

We employed a pre-post quasi-experimental design using mixed methods to assess a GANC model (Lea Mimba Pregnancy Clubs) at six health facilities in Kakamega County, Kenya. Between April 2018 and January 2019, we tracked 1652 women assigned to 162 GANC cohorts. Using an intention-to-treat approach, we conducted baseline (*N* = 112) and endline surveys (*N* = 360) with women attending immunization visits to assess outcomes including experience of care, empowerment and self-efficacy, knowledge of healthy practices and danger signs, and practice of healthy behaviors, including ANC retention. At endline, we conducted 29 in-depth interviews (IDIs) and three focus group discussions with women who were currently and previously participating in GANC, and 15 IDIs with stakeholders.

**Results:**

The proportion of survey respondents with knowledge of three or more danger signs during pregnancy more than tripled, from 7.1% at baseline to 26.4% at endline (OR: 4.58; 95% CI: 2.26–10.61). We also found improvements in women’s reports about their experience of care between baseline and endline, particularly in their assessment of knowledge and competence of health workers (OR: 2.52 95% CI: 1.57–4.02), respect shown by ANC providers (OR: 1.82, 95% CI: 1.16–2.85), and women’s satisfaction with overall quality of care (OR: 1.62, 95% CI: 1.03–2.53). We saw an increase from 58.9% at baseline to 71.7% at endline of women who strongly agreed that they shared their feelings and experiences with other women (OR: 1.73, 95% CI: 1.1–2.7). The mean number of ANC visits increased by 0.89 visits (95% CI: 0.47–1.42) between baseline (4.21) and endline (5.08). No changes were seen in knowledge of positive behaviors, empowerment, self-efficacy, and several aspects related to women’s experience of care and adoption of healthy behavior constructs. Qualitatively, women and stakeholders noted improved interactions between health providers and women, improved counseling, increased feelings of empowerment to ask questions and speak freely and strengthened social networks and enhanced social cohesion among women.

**Conclusions:**

GANC offers promise for enhancing women’s experience of care by providing improved counseling and social support. Additional research is needed to develop and test measures for empowerment, self-efficacy, and experience of care, and to understand the pathways whereby GANC effects changes in specific outcomes.

**Supplementary Information:**

The online version contains supplementary material available at 10.1186/s12913-022-08200-1.

## Background

Significant disparities remain in utilization of services and in the quality of care women receive during pregnancy and childbirth in low- and middle-income countries (LMICs). Increasing access to and utilization of high-quality antenatal care (ANC) is a key strategy in reducing maternal mortality and is associated with increased facility-based delivery [1–4] and postnatal care [5, 6]. ANC visits provide screening and detection of early signs of disease, promote healthy behaviors, and link women to services necessary for a healthy pregnancy [7]. While most women in LMICs attend at least one ANC visit (85%) [8], only 62% [9] receive the World Health Organization (WHO) previously recommended minimum of four visits, and new guidelines issued in 2016 recommend at least eight visits [10]. Access to and utilization of ANC services remain low not only because of socioeconomic inequalities, but also due to problems with infrastructure, poor quality of care, and cultural barriers [11]. A synthesis of 85 qualitative studies found that costs associated with visits, lack of privacy, limited time spent with providers, and disrespectful care kept women from attending ANC visits [12]. One study in western Kenya found that the main barriers for using ANC were negative attitudes of clinic staff, long waiting times, and costs related to services and transportation [13].

A systematic scoping analysis showed that women desire a pregnancy experience that includes emotional and social support from health providers and their communities [11]. The WHO Quality of Care Framework for Maternal and Newborn Health further recognizes respectful care, emotional support, and effective communication as important elements in women’s experience of care [14]. New models of care are needed to better respond to the needs of women for social support and improve coverage and quality of ANC. Introduced in high-income countries (HICs) as an innovative model for delivering quality care, group ANC (GANC) consists of regular meetings among small groups (7–12 women), usually of similar gestational age, facilitated by one or two skilled health providers who guide participants through clinical assessments, education activities, and support groups [15]. In HICs, GANC has been associated with higher satisfaction with care, increased social support, and enhanced relationships with providers [16–20]. Group care models have been introduced in several LMICs and demonstrated positive associations in knowledge of danger signs and adoption of healthy behaviors, such as birth preparedness, delivery in a health facility, breastfeeding initiation, and uptake of family planning [15, 21–24]. Other studies [25–27] reported the establishment of social bonds between women participating in GANC and improved relationships between women and health providers. Only one study examined the effect of GANC on empowerment, with mixed results [25].

Despite its promise, rigorous studies of the effects and women’s experience of GANC have not been conducted, and WHO has recommended further study [14]. While evidence from HIC suggests that GANC increases satisfaction with care [28], this association has not been well studied in sub-Saharan Africa. In addition, only a few studies have explored the effects of GANC on ANC retention, namely studies in Kenya, Nigeria, Malawi, and Tanzania [25, 29], where there were reported improvements. Evidence on social support is also limited, although research shows that continuous social and emotional support in childbirth affects outcomes for the mother and the newborn, including reductions in cesarean sections, antenatal hospital admissions, and mean number of hospitalization episode [28], and is protective against postpartum depression [30].

The objectives of our research study were to assess the effects of GANC on women’s experience of care and practice of healthy behaviors in pregnancy. We posit that if we implement person-centered GANC, then women will have improved knowledge of healthy behaviors and danger signs, and increased social support, which then will lead to an improved experience of care, increased self-efficacy and empowerment. and a higher likelihood of adopting healthy behaviors such as ANC retention and making birth preparations.

## Methods

We employed a pre- post quasi-experimental design using mixed methods to assess the Lea Mimba Pregnancy Clubs, a GANC model co-developed using human-centered design approaches at six health facilities in Kakamega County, Kenya. We implemented cross-sectional surveys of women seeking postnatal care or immunization up to 3 months after delivery at study sites. We used the intention-to-treat approach as the implementation plan was for all antenatal services to be provided in the group format.

### Study setting

With a maternal mortality ratio of 362 per 100,000 live births and a neonatal mortality rate of 22.2 per 1000 live births, Kakamega County lags behind the rest of the country [31]: only 45% of women receive at least four ANC visits, as recommended in national guidelines; just over 20% receive any care during the first trimester of pregnancy; and slightly less than half (47%) deliver with a skilled birth attendant, versus a national average of 61% [31].

Lea Mimba (Taking Care of Your Pregnancy) Pregnancy Clubs were implemented in six health facilities representing urban, peri-urban, and rural sites in the Lurambi and Malava sub-counties of Kakamega County, specifically one urban referral hospital (level 5), one peri-urban county hospital (level 4), and four peri-urban or rural health centers (level 3). The latter are staffed by midwives or nurses and clinical officers and provide inpatient and outpatient curative and preventive services. County hospitals offer these as well as specialized services (such as cesarean sections and x-rays), and county referral hospitals provide additional advanced care. Monthly ANC visits in the six facilities ranged from 127 to 787, and the number of ANC providers per facility ranged from 5 to 18 in April 2018.

### Intervention design

In collaboration with the national and county health governments, the Kenya Progressive Nurses’ Association (KPNA), and Scope (formerly M4ID), which specializes in human-centered design, we co-designed the Lea Mimba Pregnancy Clubs with women, health care providers, and health officials. Eight to ten women of similar gestational age met with the same health provider during sessions based on the WHO-recommended eight-visit model [10] and national standards. Women and health providers discussed a range of health topics including recognition of danger signs, care of the newborn, family planning, among others. Sessions supported interactive learning and enabled discussion of challenges and problem-solving with peers. Rituals, such as opening and closing activities and singing, were used to create a sense of membership and solidarity. Women were paired to take measurements, such as weight and blood pressure and to remind each other for future appointments, to strengthen their feelings of empowerment and solidarity.

Nurses and midwives received training and mentorship on the new model of care. At the initial ANC (booking) visit, pregnant women chose whether to enroll in Lea Mimba and were assigned to a group based on their estimated delivery date. It was expected that facilities would transition to only providing GANC and enrolling all pregnant women into GANC. Facility staff sent phone reminders for pregnancy club sessions in advance. Study-specific registers were used to track women over time. Community health volunteers (CHVs) also supported follow-up for group appointments and encouraged women to attend ANC through community outreach.

### Study sample

At baseline, women were interviewed before commencing GANC, and at endline, regardless of GANC participation, all women who met the eligibility criteria were interviewed. We sampled 112 respondents at baseline and 360 at endline and removed the 2 women whose age was missing from age-disaggregated data. In addition, from April 2018 to March 2019, data on age and number of ANC visits attended, were extracted from study registers and facility registers to track women attending group and individual ANC sessions across the six facilities. Women were included in the analysis if they met either of two criteria: (1) no one in the group had reported a visit more recent than January 31, 2019 (data was available through March), or (2) at least one member of the group had completed eight ANC visits. Only women in completed cohorts were included.

For the qualitative component, at endline, we conducted 44 in-depth interviews (IDIs) and three focus group discussions (FGDs) using semi-structured interview guides developed for this study (included as Additional File 1). Participants were selected purposefully using maximum variation sampling to explore and understand experiences of the intervention from a variety of perspectives. Sites were selected purposively based on varying outcomes of ANC initiation and retention [32]. From the selected sites, twenty-nine IDIs were conducted with women who had delivered and completed at least four GANC visits, women who had delivered but did not complete four GANC visits, and women currently in GANC who had completed four GANC visits. Across these categories, the sample was designed to include women of different age groups, theorizing that they would experience the care differently. We also conducted 15 IDIs with key stakeholders, including county and facility health managers, health workers providing GANC, and CHVs. Three FGDs with five to eight unique participants each were conducted with adolescents and women who did not complete four GANC visits across the six health facilities. FGDs were used for this population to encourage women to open up about this sensitive topic. Saturation was achieved before completing the proposed sampling; however, interviews and FGDs continued, anticipating that different experiences might yield new insights.

### Data collection and analysis

The study used quantitative and qualitative measures to assess changes in key outcomes (Table 1). All interviews were conducted in English or Kiswahili. Five persons on the research team read through and analyzed the results.

**Table 1:** Key outcomes and respective measures

Outcome	Quantitative Methods	Qualitative Methods
Knowledge	Cross-sectional surveys at baseline and endline included an open-ended question for clients to identify: • danger signs/complications • how to improve own and baby’s health Data collectors compared the client’s response to a list of expected responses based on the educational content offered in ANC. Data was analyzed using logistic regression.	IDIs with women currently in GANC IDIs with women who completed at least 4 GANC visits IDIs with women who did not attend at least 4 GANC visits FGDs with women who completed GANC FGDs with women who did not complete 4 group ANC sessions IDIs with health providers, facility managers, CHVs, and county health team Questions focusing on: What learning has made a difference in your life, if any Likes and dislikes about participating in or implementing pregnancy club
ANC experience of care (based on WHO framework: effective communication, respect and dignity, and emotional support)	Cross-sectional surveys at baseline and endline included a question on the below topics for the client to rate using a Likert scale. • Sharing feelings and experiences with other women (social support) • Knowledge and competence of health workers • Respect shown to respondent by ANC providers • Trust in ANC providers • Language ANC providers used toward respondent • Information and counseling provided about pregnancy, delivery, and postnatal care • Overall quality of care Responses were transformed into a binary response for the purpose of hypothesis testing, as the data did not meet the proportional odds assumption of ordinal regression. For “sharing feelings and experiences with other women,” we compared women who responded they “strongly agree” to those who did not due to the heavily skewed results. For all other measures, we compared women who rated the characteristic of experience as “excellent” or “very good” to those who did not. Data was analyzed using logistic regression.	IDIs with women currently in GANC IDIs with women who completed at least 4 GANC visits IDIs with women who did not attend at least 4 GANC visits FGDs with women who completed GANC FGDs with women who did not complete 4 group ANC sessions IDIs with health providers, facility managers, CHVs, and county health team Questions focusing on: Likes and dislikes about participating or implementing pregnancy club Describe relationships between women and with health providers Benefit of participating in pregnancy clubs
Empowerment	Cross-sectional surveys at baseline and endline included a series of questions related to pregnancy-related empowerment, defined by Patil et al. as “the quality of communication and connectedness pregnant women feel with their care providers and peers, their participation in decision-making, and their capacity to recognize and engage in pregnancy-related healthy behaviors.” Each individual question was collected using a Likert scale, with a point-value attached to each response. The sum of these point-values was used to calculate an overall PRES score for each client. Data was analyzed using logistic regression.	IDIs with women currently in GANC IDIs with women who completed at least 4 GANC visits IDIs with women who did not attend at least 4 GANC visits FGDs with women who completed GANC FGDs with women who did not complete 4 group ANC sessions IDIs with health providers, facility managers, CHVs, and county health team Questions focusing on: Benefit of participating in pregnancy clubs Likes/dislikes about pregnancy club – open-ended question that revealed knowledge improvements
Adoption of healthy behaviors	Cross-sectional surveys at baseline and endline included a question on the following topics. • Birth preparations: open-ended question for clients to identify preparations they had made, and project technical staff compared the client’s response to a list of expected preparations based on the educational content offered in ANC The following information was extracted from health facility registers: • Retention: number of ANC visits by an ANC client Data was analyzed using logistic regression, except for retention, which was analyzed using linear regression.	IDIs with women currently in GANC IDIs with women who completed at least 4 GANC visits IDIs with women who did not attend at least 4 GANC visits FGDs with women who completed GANC FGDs with women who did not complete 4 group ANC sessions IDIs with health providers, facility managers, CHVs, and county health team Questions focusing on: Benefit of participating in or implementing pregnancy clubs Likes/dislikes about pregnancy clubs

#### Quantitative research

Table 1 describes the quantitative and qualitative measures used for our key outcomes. Questions about basic demographics and background information were drawn from the Demographic and Health Survey [31], and questions about complications during pregnancy and knowledge about complications, as well as birth preparedness, were developed based on a Ghana GANC study [23]. Questions about experience of care were modified from a non-licensed tool developed for a study on respectful care/disrespect and abuse during delivery in Tanzania [33]. For our measure of empowerment, we used the Pregnancy-Related Empowerment Scale (PRES), a non-licensed tool previously used in Tanzania and Malawi [25] that defines empowerment as “the quality of communication and connectedness pregnant women feel with their care providers and peers, their participation in decision-making, and their capacity to recognize and engage in pregnancy-related healthy behaviors (p. 34).” For our study, we adapted the response options used to comprise the PRES score from a four-point to a five-point Likert scale. We defined experience of care based on the three elements outlined in WHO’s framework for the quality of maternal and newborn health care [14]: effective communication, respect and dignity, and emotional support. We measured these elements quantitatively.

We analyzed all quantitative outcomes from facility registers and client surveys using univariate and multilevel logistic regression models for binary outcomes and multilevel linear regression models for continuous outcomes, allowing facility-level clustering to be accounted for in measures of uncertainty. In line with the emerging consensus in the statistics literature, we present statistical measures of uncertainty as continuous, rather than dichotomizing these results into significant or not significant [34, 35]. Multilevel models with random effects and random slopes were used to meet the assumption of independence, except where this produced a singular result, in which case facilities were removed as clusters from the model as needed. None of the hypothesis tests conducted use multiple predictors and therefore meet the multicollinearity assumption of logistic regression. No corrections were applied for making multiple comparisons, so secondary and intermediate objectives should be interpreted with greater caution. All quantitative analysis was conducted using R version 3.5.1 [36].

#### Qualitative research

IDI and FGD guides were first developed by two of the authors for a study in Uganda and then modified for the Kenya context by other authors, translated into Kiswahili as needed, and pilot tested. They were designed to elicit information about experience with GANC, primarily among women and providers, including what they thought about the clubs, how the clubs affected their lives, how they talk about the clubs with others, and ease of participating in/providing GANC.

FGDs and IDIs were facilitated by six consultants (four female, two male) who had no previous relationship with the participants because the authors were either known to the participants or unable to conduct interviews in Kiswahili. The lead consultant held a doctorate degree, while the others had received a diploma or undergraduate degree. The consultants completed a two-day training covering research ethics, IDI/FGD guides, and other key aspects, such as interviewer bias. Consultants contacted women and health providers to participate by phone; some were unreachable or unavailable. Some refusals were due to husband’s not allowing permission and adolescents were particularly difficult to reach. Women were interviewed in a private community location, while other stakeholders were interviewed in private rooms at their work. Participants were reimbursed for transport costs. IDIs and FGDs were conducted in Kiswahili or English and audio recordings were then transcribed and Kiswahili transcripts translated into English supported by field notes. IDIs ranged from 45 to 75 minutes and FGDs were 60–90 minutes.

We used thematic analysis, starting with a codebook developed from the main concepts in the interview guides and adapting the codebook based on reading of transcripts and joint coding of two transcripts. We double-coded 10% of the transcripts in Dedoose. Findings for each code were summarized and placed in a matrix comparing respondents for each code. Three authors discussed the frameworks to agree on emerging themes and patterns.

### Ethical approval

Ethical approval was obtained from the Jaramogi Oginga Odinga Teaching and Referral Hospital Ethical Review Committee. Each participant provided written consent before taking part in the study.

## Results

### Description of the sample

Between April 2018 and January 2019, 1652 women were enrolled in group ANC, out of 5120 new ANC clients recorded in the national health management information system for the study sites. Of the 162 groups formed, 103 were completed at the time of data extraction, with a total of 1145 women (Table 2). The mean number of women per group was 11.1. We conducted more qualitative interviews with women aged 20–24 who participated in GANC and fewer with adolescents (as they were harder to access) and interviewed more female than male stakeholders.

**Table 2:** Characteristics of respondents

**GANC Facility Registry**			
Age	*N* = 1650 (%)		
10–14	1 (0.06%)		
15–19	287 (17.4%)		
20–24	593 (35.9%)		
25–34	656 (39.8%)		
35+	113 (6.8%)		
Facility	*N* = 1652 (%)	GANC cohorts completed (103)	
Level 3	388 (23.5%)	19	
Level 3	218 (13.2%)	13	
Level 3	254 (15.4%)	19	
Level 3	230 (13.9%)	16	
Level 4	350 (21.2%)	23	
Level 5	212 (12.8%)	13	
**Survey Respondents**			
	Baseline (*N* = 112)	Endline (*N* = 360)	
Age			
15–19	13 (11.6%)	39 (10.8%)	
20–24	49 (43.6%)	97 (26.9%)	
25–34	45 (40.2%)	201 (55.8%)	
35+	5 (4.5%)	23 (6.4%)	
Number of lifetime births			
1 birth	44 (39.3%)	93 (25.8%)	
2 births	30 (26.8%)	106 (29.4%)	
3 births	20 (17.9%)	79 (21.9%)	
4 births	11 (9.8%)	43 (11.9%)	
5 births	2 (1.8%)	22 (6.1%)	
6+ births	5 (4.5%)	17 (4.8%)	
Marital status			
Never married	18 (16.1%)	60 (16.7%)	
Currently married	81 (72.3%)	286 (79.4%)	
Separated	4 (3.5%)	3 (0.8%)	
Divorced	0 (0.0%)	4 (1.1%)	
Widowed	0 (0.0%)	2 (0.6%)	
Partnered— living together	6 (5.4%)	1 (0.3%)	
Partnered— not living together	3 (2.7%)	3 (0.8%)	
N/A	0 (0.0%)	1 (0.3%)	
Highest level of education			
None	3 (2.7%)	8 (2.2%)	
Before Primary	9 (8.0%)	3 (0.8%)	
Primary	38 (33.9%)	139 (38.6%)	
Vocational	4 (3.6%)	3 (0.8%)	
Secondary	40 (35.7%)	160 (44.4%)	
Training Post-Secondary	7 (6.3%)	28 (7.8%)	
University	11 (9.8%)	19 (5.3%)	
Years of living in village or town			
< 1 year	1 (.9%)	25 (6.9%)	
1–2 years	47 (42.0%)	90 (25.0%)	
3–5 years	25 (22.3%)	111 (30.8%)	
6–10 years	16 (14.3%)	72 (20.0%)	
11–20 years	17 (15.2%)	45 (12.5%)	
21–30 years	5 (4.5%)	15 (4.2%)	
> 30 years	1 (0.9%)	2 (0.6%)	
Religion			
Muslim	2 (1.8%)	5 (1.4%)	
Christian	97 (86.6%)	353 (98.1%)	
Traditional	12 (10.7%)	2 (0.6%)	
None	1 (0.9%)	0 (0.0%)	
Number of household members			
< 3	4 (3.6%)	10 (2.8%)	
3–4	54 (48.2%)	147 (40.8%)	
5–6	30 (26.8%)	131 (36.4%)	
7–8	18 (16.1%)	49 (13.6%)	
> 8	6 (5.4%)	23 (6.4%)	
Head of household by gender			
Man	98 (87.5%)	323 (89.7%)	
Woman	10 (8.9%)	35 (9.7%)	
Do not know	4 (3.6%)	2 (0.6%)	
Qualitative Respondents			
In-depth interviews (*N* = 29)			
Age	Women who have delivered and competed at least 4 group ANC visits (*N* = 20)	Women who have delivered but did not complete 4 visits (*N* = 6)	Women who are currently in group ANC and have completed 4 group visits (*N* = 3)
< 20	8	2	1
20–24	8	2	2
25+	4	2	–
Facility level			
Level 3	10	6	2
Level 4	5	–	–
Level 5	5	–	1
Focus Group Discussion (*N* = 19)			
Group	Number of Participants		
Young women (age 20–25)	8		
Adolescents (15–19)	6		
Older women (26+)	5		
Stakeholder IDI (*N* = 15)			
Position			
Health facility manager	4 (male = 1; female = 3)		
Health care provider	4 (male = 1; female = 3)		
CHVs	4 (male = 1; female = 3)		
County health official	3 (male = 1; female = 2)		

Overall, the demographic changes between the baseline and endline samples were less than 10%, aside from a few exceptions listed here. At baseline, 44% of the sample was aged 20–24, which declined to 27% at endline, and in the 25–34 age group, these proportions were 40 and 56%, respectively. The overall pattern for lifetime number of births was similar for two births and higher, but the percentage of primigravidae was lower at endline (25.8%) compared to baseline (39.2%). A higher proportion of baseline (42.0%) than endline (25.0%) respondents reported living in their current town for only 1–2 years. 10.7% of baseline respondents reported traditional religion compared to 0.6% at endline, driving an increase from 86.6 to 98.1% reporting Christian religion. At endline, only 36.1% of the sample had participated in GANC.

### Outcomes

Table 3 outlines the outcomes that we measured quantitatively from survey data and the themes that emerged from the FGDs and IDIs in terms of knowledge, experience of care, empowerment and self-efficacy, and adoption of healthy behaviors.

**Table 3:** Summary of results

	Percentage		Odds Ratio (95% CI)
	Baseline (***N*** = 112)	Endline (***N*** = 360)	
**Knowledge**			
Women who could identify 3 or more danger signs of complications during pregnancy	7.1%	26.4%	**4.58 (2.26–10.61)**
Women who could identify 3 or more things a woman can do during pregnancy to improve her and her baby’s health	30.4%	37.5%	1.37 (0.87–2.19)
Qualitative themes: Knowing the why and not only the what; practical tips and information; mutual learning for women and health providers			
**ANC experience of care: Percentage rating “excellent” or “very good” based on 5-point Likert scale**			
Women who strongly agreed that they shared their feelings and experiences with other women	58.9%	71.7%	**1.73 (1.1–2.7)**
Knowledge and competence of health workers	57.2%	78.6%	**2.52 (1.57–4.02)**
Respect shown to respondent by ANC providers	59.8%	73.3%	**1.82 (1.16–2.85)**
Experienced disrespect and humiliation	7.1%	9.7%	1.40 (0.66–3.33)
Trust in ANC providers	58.1%	65.0%	1.23 (0.78–1.91)
Language ANC providers used toward respondent	57.2%	65.6%	1.39 (0.88–2.16)
Level of privacy and confidentiality observed during ANC	55.3%	62.0%	1.29 (0.79–2.22)
Intent to use same facility in a subsequent pregnancy	88.8%	93.2%	1.87 (0.39–9.47)
Very likely to recommend facility to other women	75%	90.8%	2.82 (0.39–9.47)
Overall quality of care	56.3%	68.3%	**1.62 (1.03–2.53)**
Qualitative themes: Sharing experiences to solve problems, giving each other strength and encouragement to cope, feeling that nurses create an open and safe space			
**Empowerment and self-efficacy: Percentage who “strongly agree” based on a 5-point Likert scale**			
You could ask your ANC provider about your pregnancy.	67.0%	63.1%	0.86 (0.54–1.36)
Since you began antenatal care, you have been making more decisions about your health.	74.1%	74.7%	1.02 (0.61–1.66)
You felt you had a right to ask questions when you don’t understand something about your pregnancy.	83.0%	76.4%	0.67 (0.37–1.16)
You were able to change things in your life that are not healthy for you or the baby.	75.0%	78.3%	1.21 (0.73–1.99)
You did what you could do to have a healthy baby.	92.9%	87.5%	0.54 (0.23–1.12)
You could talk to your partner about your pregnancy and planning for delivery.	85.6%	76.9%	0.56 (0.21–1.23)
Qualitative themes: Feelings of self-efficacy			
**Adoption of healthy behaviors**			
Number of ANC visits	4.21	5.08	**95% CI of difference: 0.47–1.42 visits**
Number of ANC visits among under 25 years of age	4.23	5.11	**95% CI of difference: 0.27–1.34 visits**
**Birth preparations**			
Women reporting that they made 2 or more of any of the listed preparations	33.0%	48.9%	**1.94 (1.24–3.05)**
Women reporting that they prepared items for the baby or delivery	64.3%	71.9%	1.61 (0.94–2.72)
Qualitative themes: Making a difference for the better			

#### Knowledge

We assessed changes in knowledge from survey data, interviews, and FGDs. The proportion of survey respondents able to identify three or more danger signs of complications during pregnancy more than tripled, from 7.1% at baseline to 26.4% at endline (OR: 4.58; 95% CI: 2.26–10.61) (Table 3). Similarly, the percentage of women who could identify three or more ways to improve their and their baby’s health increased from 30.4 to 37.5% (OR: 1.37; 95%CI: 0.87–2.19)); however, the 95% confidence interval suggests that this change may be due to chance (Table 3).

In qualitative interviews and discussions, women described changes in knowledge as a result of Lea Mimba, in particular gaining practical information on how to care for themselves and their baby. Women reported that they not only learned essential information but also understood better why what they were doing was important for their health and for their baby and that this deeper understanding made them more willing to adopt healthy behaviors.

Even that part of taking drugs... we never knew the importance of taking these drugs … we would say the drugs are bad, they make someone nauseated when you take them. We were taught the importance of the drug that makes the baby grow well in the uterus … . Nowadays I can’t miss taking it. *Adolescent, county hospital*

Women described learning not only from health providers but also from peers. Providers also described a mutual learning environment where they gained insights into cultural practices and beliefs, which helped them understand women’s situations. As a result, they were able to provide better counseling and communication.

To me personally it has opened my eyes, the interaction with these mothers has taught me a lot, we teach each other actually, because there are some things they know that we never knew; some things are taboo actually, so you try to know misconceptions so you try to rectify [them] and they take it positively. *Health provider, county hospital*

#### Experience of care

We found improvements in women’s reports about their experience of care between baseline and endline, particularly in knowledge and competence of health workers (OR: 2.52 95% CI: 1.57–4.02), respect shown by ANC providers (OR: 1.82, 95% CI: 1.16–2.85), and women’s satisfaction with overall quality of care (OR: 1.62, 95% CI: 1.03–2.53) (Table 3). We saw an increase from 58.9% at baseline to 71.7% at endline of women who strongly agreed that they shared their feelings and experiences with other women (OR: 1.73, 95% CI: 1.1–2.7). We did not find any evidence that intent to use the same facility in a subsequent pregnancy changed between baseline and endline or that the proportion of respondents self-described as “very likely” to recommend the facility to other women changed. Similarly, we did not find evidence of changes in reported disrespect or humiliation.

Through qualitative methods, women reported an improved experience of care in GANC as compared to traditional ANC—including improved communication, feelings of respect and dignity, and social and emotional support and solidarity.

Our service provider was very good. She was very free and open and in any case you had any problem and you are pregnant, you could still approach her and she would teach you. *Young woman, county hospital*

GANC participants described the social support, trust, and solidarity they gained by sharing experiences and giving each other strength and encouragement to cope. They described receiving support that was both practical, such as sharing transport, as well as emotional, such as dealing with the stress of a pregnancy complication. Most women described forming bonds with at least some of the women in their group and with the health provider. Discussions with their peers enabled them to solve problems together.

They are friends. When one tells her experience and another also talks about her experience, they help to sort out the problem... When one woman does not come, her friend will remind her of the next meeting, and she will make an effort of looking for her and asking her why she has not seen you. *CHV, health center*

Women valued these aspects of GANC and talked about how they maintained the relationships even outside the group sessions. A number of women talked about how the relationships would likely continue after the pregnancy. A few expressed disappointment when the health provider who was facilitating their sessions changed and was replaced by another, which may indicate that the women had developed a bond with the provider. Health providers also seemed to gain some satisfaction from developing closer relationships with women and found it helped them provide better quality of care. In particular, women noted improved respectfulness from the health provider and a reduction in perceived discrimination. Adolescents in particular reported being treated more respectfully and felt at ease, free from discrimination and judgement.

Lea Mimba really encouraged mothers; when we used to attend, most of the nurses were friendly. In normal ANC clinics, you will find some nurses don't attend to you well, but in the Lea Mimba club, the nurses did not discriminate against anyone. When you go to other clinics you are told you are dirty, here you are attended to the way you are. *Adolescent, county hospital*

#### Empowerment and self-efficacy

We did not find evidence of changes in empowerment, as measured through PRES score, between baseline and endline in quantitative data, but women in qualitative interviews and discussions, especially adolescents, described increasing feelings of self-efficacy and confidence to adopt more healthy behaviors. Adolescent women reported that they became more empowered to do things they previously felt they could not do.

Yes, for me I never imagined I could take care of my pregnancy, I never saw myself taking care of a child and using family planning, I thought it was a lot of work. But after the Lea Mimba lessons, I can do all these things. *Adolescent, referral hospital*

For the groups in general, health providers described how women were more active in taking a role in their ANC experience, such as asking for services or tests, as expressed by this provider:

They really liked it [group ANC] and if you had not taken their pressure, they are the ones who would remind you that sister you have not taken my pressure, teacher you have not weighed me. We used to teach them how to do some of these things … unlike the normal ANC where a mother walks in and you are the one who does everything for her, but now they are the ones doing these things for themselves. *Health provider, health center*

#### Adoption of healthy behaviors

**Fig. 1:**
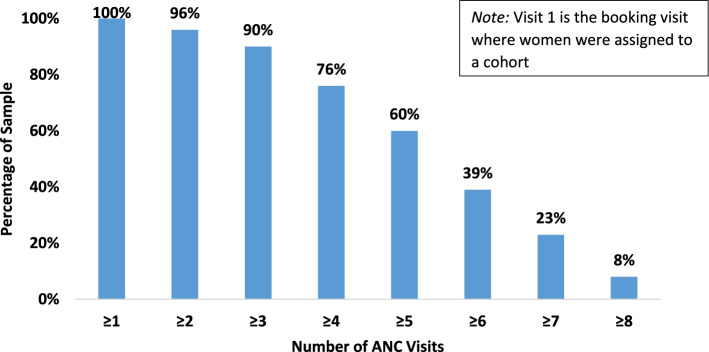
Number of ANC sessions attended by women enrolled in group ANC

The mean number of ANC visits increased by 0.89 visits (95% CI: 0.47–1.42) between baseline (4.21) and endline (5.08). Among women under 25, the mean number of visits increased by 0.79 (95% CI: 0.27–1.34) between baseline (4.23) and endline (5.11). There was no evidence from client surveys that the reason women attended ANC changed over the course of implementation. Figure 1 shows the retention of women who were enrolled in GANC: 96% of women enrolled during ANC1 attended at least one more ANC (group or individual), 76% attended at least four visits, and 8% attended eight.

The proportion of women reporting two or more of any of the listed preparations (Table 4) increased from 33.0% at baseline to 48.9% at endline (OR: 1.94; 95% CI 1.24–3.05); however, the improvement of 7.9% in preparing items for the baby or delivery may be due to chance (OR: 1.61; CI: 0.94–2.72). In qualitative interviews and discussions, both women and health providers noted improved behaviors in preparing for childbirth. They reported buying items for the baby, saving money for transport once labor began, and packing a bag to take to the facility.

At least nowadays they come when they are prepared, they carry clothes for the baby, and she has a towel to wrap the baby, so I think that it has improved [behaviors]. *Health facility manager, health center*

my first pregnancy... I did not save money to buy clothes for the baby and transport costs before the baby was delivered. But for this one, I was taught and I prepared myself early. I bought the baby’s clothes early and saved some cash for delivery costs. *Older woman, county hospital*

In addition, women across all age groups and district and health facility staff described how participating in Lea Mimba helped women adopt positive behaviors for a healthy pregnancy and newborn baby. In particular, young and adolescent women indicated that the advice and information helped them make improvements and had a positive effect on their lives.

These sessions really helped me, because I was opting to abort but after the sessions I did not abort. Then I did not know anything like taking care of my pregnancy, but through attending the sessions I survived with the pregnancy. *Adolescent, health center*

And then you should not bathe the baby but just wipe, just wipe until the umbilical cord drops off. Let it heal, that is when you can start bathing her in much water. I did that but for those other ones I used to bathe them immediately and it used to take time for the umbilical cord to heal, so it was different for this other one. Those lessons really helped me. *Older woman, health center*

**Table 4:** Responses to question: What preparations did you make?

Response	Baseline (***N*** = 112)	Endline (***N*** = 360)
Saved money	56 (50%)	201 (55.8%)
Selected facility	4 (3.6%)	24 (6.7%)
Arranged transport	5 (4.5%)	50 (13.9%)
Prepared items for the baby/delivery	72 (64.3%)	259 (71.9%)
None	19 (17%)	44 (12.2%)
Do not know	1 (0.9%)	0 (0%)
Other	0 (0%)	6 (1.7%)
Not applicable	1 (0.9%)	1 (0.3%)

*Note:* Multiple responses were allowed

## Discussion

Our study found GANC is associated with enhanced social support from other women and from health care providers, with some evidence for (1) improved knowledge, (2) improved experience of care, (3) enhanced empowerment and self-efficacy, and (4) adoption of healthy behaviors. These findings suggest that our understanding of the pathways through which GANC affects outcomes may be incomplete, and further research may be needed to generate new hypotheses. Our inconsistent results can be explained by several factors: a short implementation period (10 months) to transition ANC services at project sites to GANC and observe population-level effects; low adoption of GANC in our survey sample (while we intended for all ANC clients to shift to GANC, at endline only 36.1% had participated in GANC); and limitations in some of our quantitative measures (see next section). The uptake of the intervention took longer than expected, and as a result less women have been exposed to the intervention given the duration of pregnancy and the aim to survey women who had completed their pregnancy. Nonetheless, given the changes observed, we expect that the GANC intervention had spillover effects that may have also changed the experience of individual ANC interactions.

The evidence for improvements in knowledge was inconclusive: while knowledge of danger signs tripled among GANC participants, no statistical difference was found in knowledge of positive health behaviors between baseline and endline. This contradicts what is generally found in the literature [17, 21, 37, 38], which has reported improvements in all aspects of knowledge. A recent study in Nepal [39] also found that knowledge of danger signs improved, but knowledge of birth preparation decreased, from baseline to endline. Our qualitative data revealed that participating women identified learning as the aspect they most valued in GANC; they appreciated the tips and information and an understanding of the “why” in addition to the “what.” We found similar findings elsewhere in the qualitative literature: a study in the United States [20] reported how women spoke of understanding, rather than just learning, information; and studies in Rwanda and Bangladesh [26, 27] reported that women valued the improved knowledge they gained through interacting with others and in new ways. The lack of a change in knowledge of positive health behaviors needs further exploration—perhaps our curriculum emphasized recognition of danger signs over self-care, or our measure needs to be revised.

To date, there are no published studies from LMICs that quantitatively measure experience of care from GANC. Most of the literature on GANC in HICs has studied effects of GANC on satisfaction of care and found an increase [18–20] with the exception of one trial [17] in which the evidence for the mean difference was weak. In our study, women reported improvements in knowledge and competence of health workers, respect shown by ANC providers, and women’s ratings of overall quality of care. However, we found no change in other elements of women’s experience, such as recommending the facility to other women, information and counseling from health providers, and the rate at which women reported experiencing disrespect or humiliation. Our qualitative data indicates that GANC may have supported more effective communication. Some women described their experiences as nondiscriminatory and respectful and felt sessions provided an open and safe space to discuss questions and concerns. These discrepancies in women’s experience of care may be due to cultural understanding of terms and/or social or courtesy biases.

While our study found mixed results of the effect of GANC on supporting effective communication and providing respectful and dignified care, we found strong evidence of the effect of GANC in providing women with social support. Club members described developing bonds with health providers and with other women that fostered trust, enabling them to jointly solve practical and emotional problems and cope with pregnancy stresses. Health care providers also noted an improvement in their relationships with women. Studies in LMICs confirm this result: women valued the peer support and improved relationships with health providers and the support that came from these bonds [25–27, 40, 41]. This finding was perhaps due to the emphasis of our GANC model on fostering social bonds by linking women with other pregnant women at similar gestational ages, pairing individual women, and using interactive learning techniques to help women discuss problems and challenges.

We hypothesized that improved knowledge and social support would contribute to feelings of empowerment and self-efficacy among women participating in GANC; however, we found no difference in quantitative measures between baseline and endline for empowerment. We used a scale previously validated in Malawi and Tanzania but were unable to validate this in the county context prior to the study, which may explain this finding. The only quantitative study to date assessing the effect of GANC on women’s empowerment [25] had mixed results: women in GANC had higher empowerment scores in Malawi but not in Tanzania. We found only one qualitative study [39] assessing empowerment as a result of GANC, where women reported feeling empowered to speak up in a group setting. In our qualitative data women, most notably adolescents, reported feelings of empowerment and self-efficacy to do things they felt they could not before they attended GANC. Health providers also described how women were more active in their ANC experience, and the shifted power dynamics may have been empowering for all. Our findings and the lack of literature in this area point to the need for further research.

We observed positive qualitative and quantitative results in one health-seeking behavior, birth planning: respondents were almost twice as likely to have made two birth preparations at endline compared to baseline. In our qualitative data, women reported practicing healthy behaviors, such as taking nutritional supplements and setting aside money for delivery costs. Studies in Ghana [23] and Iran [22] found similar results: women in GANC were more likely to practice healthy pregnancy-related behaviors and make preparations for childbirth. In addition, we found an improvement in ANC retention. To date, few studies have assessed the effects of GANC on retention: studies in Malawi [25], Tanzania [25], Nigeria [29] and Kenya [24] reported ANC4+ retention was higher in GANC than individual ANC; a study in Nepal [39], however, found no change in ANC completion.

### Study limitations

Interpretation of our results must be contextualized within several limitations. First, pregnant women who participated in GANC opted in for this intervention, despite the intervention design, which aimed to enroll all women; thus, the study may have enrolled women who were more eager to participate in ANC. Our survey sample were women who were attending immunization and who are, therefore, more likely to seek care (e.g., facility-based delivery, family planning) and are not comparable to the general population. At baseline, the number of surveys collected from several facilities were low, which affected the ability to estimate facility-level mixed effects in our regression models. Certain subgroups, such as women aged under 15, are not well represented and were difficult to find. We rarely found information to estimate variation between clusters for sample size calculations, so our study may be underpowered. The design of our instruments focused on whether a behavior had changed or not and did not capture why people behave in a certain way or the underlying factors that affect behavior (e.g., decision-making power, money, cultural beliefs), and may have contributed to our mixed results in adoption of healthy behaviors. Our survey instrument may not have adequately captured the constructs we intended. Our short implementation period (10 months) limits our ability to see changes in our outcomes. Furthermore, we cannot make any causal inference from our study due to the quasi-experimental design. Our intervention sites were non-random, and we did not have a comparison group. We cannot be certain how generalizable our findings may be to other health facilities in Kakamega County or other contexts. Rather, our study provides a specific example and is best considered alongside other emerging research on GANC.

## Conclusion

Our findings suggest that our understanding of the pathways through which GANC affects outcomes may be incomplete. One alternative pathway to consider is that GANC may lead to changes in knowledge, social support, and experience of care, which in turn lead to improved empowerment and self-efficacy, resulting in adoption of healthy behaviors. We suggest further development of quantitative and qualitative measures to assess empowerment, self-efficacy, and experience of care, and more research on the mechanisms of change in GANC. Our research has shown that GANC improved some elements of women’s experience of care through improved counseling and social support. Traditional ANC must be transformed to provide women with high-quality standards-based care that is responsive to their needs for counseling, psychological support, and social connections with other women.

## Supplementary Information

**Additional file 1: **IDI and Focus Group for ANC clients, health providers and health facility managers.

## Data Availability

The datasets used and/or analyzed during the current study are available from the corresponding author on reasonable request.

## Abbreviations

ANC: Antenatal care
CHV: Community health volunteer
DHS: Demographic and Health Survey
FGD: Focus group discussion
GANC: Group antenatal care
HICs: High-income countries
IDI: In-depth interview
KPNA: Kenya Progressive Nurses’ Association
LMICs: Low- and middle-income countries
WHO: World Health Organization
