# Correction: “Taking care of your pregnancy”: a mixed-methods study of group antenatal care in Kakamega County, Kenya

**DOI:** 10.1186/s12913-022-08435-y

**Published:** 2022-08-18

**Authors:** Aleefia Somji, Kate Ramsey, Sean Dryer, Fredrick Makokha, Constance Ambasa, Brittany Aryeh, Kathleen Booth, Serge Xueref, Seneca Moore, Ralpher Mwenesi, Shafia Rashid

**Affiliations:** 1 Management Sciences for Health (MSH), Alexandria, USA; 2 Scope, Brooklyn, USA; 3 Globally Minded Foundation, Burgas, Bulgaria; 4 Ministry of Health, Kakamega, Kenya; 5 Independent Consultant, Kakamega, Kenya; 6 Partners in Health (PIH), Boston, USA; 7 Columbia University Mailman School of Public Health, New York, USA; 8 MSH, Lyon, France; 9 Independent Consultant, Nice, France; 10 MSH, New York, USA

**Correction to: BMC Health Serv Res 22, 969 (2022)**

**https://doi.org/10.1186/s12913-022-08200-1**

Following publication of the original article [1], it was noted that due to a typesetting mistake, an error in the Abstract wasn’t corrected.

The sentence currently reads:

F studies show associations between GANC and various outcomes.

The sentence should read:

Few studies show associations between GANC and various outcomes.

The original article [1] has been corrected. The publisher apologises to the authors and readers for the inconvenience caused by the error.
