# Supplementary material for: “Taking care of your pregnancy”: a mixed-methods study of group antenatal care in Kakamega County, Kenya
Source: BMC Health Serv Res. 2022 Jul 29;22:969. doi: 10.1186/s12913-022-08200-1 (PMC9336107; doi:10.1186/s12913-022-08200-1)
Supplement: Supplementary file 1 — Additional file 1: IDI and Focus Group for ANC clients, health providers and health facility managers. [file 12913_2022_8200_MOESM1_ESM.docx]

**Focus Group Discussions for Group ANC Clients**

Sub County_________________________________________________

Health Facility Name ______________________________________

Date ___________________________________________________

Time started: __________

Time ended___________

Interviewer/data collector / Mtafiti: __________________________________

Number of women participating in the FGD __________ (should NOT be more than 10)

**Facilitator’s welcome, introduction and instructions to participants**
Welcome and thank you for volunteering to take part in this in-depth interview. My name is __________________, and I am conducting this interview on behalf of MSH to gather feedback on the Lea Mimba sessions. You have been asked to participate as your point of view is important. Participating is voluntary. I know you are busy and I appreciate your time. You will be compensated for your time at the end of this interview.

**Introduction:** This in-depth interview is designed to assess your thoughts about your experience in the Lea Mimba club sessions in which you participated. We would like to get your thoughts so that we can improve the Lea Mimba program. The interview will last for about 45 – 60 minutes. Your participation is entirely voluntary and you can leave the group discussion at any time. I will share with you an informed consent form for you to sign. We will be recording this session and taking notes so that we can capture everything you are saying. None of this data will have names or any personal information associated with it.

*(SECURE INFORMED CONSENT)*

Before we start, do you have any questions?

**Ground rules.** The most important rule is that only one person speaks at a time. There are no right or wrong answers.

- When you do have something to say, please do so. There are many of you in the group and it is important that I obtain the views of each of you. I would ask if you can all check yourself too – if you feel as if you are speaking too much take a step back and lets others speak. If you feel as if you are not speaking enough, step in and speak some more.
- You do not have to agree with the views of other people in the group.
- No one should discuss any information shared by anyone in the group with anyone outside the group.  All the information discussed is confidential and should not be shared with others outside the group.
- Does anyone have any questions?
- No one is forced to participate in this discussion. Do you all agree to participate?
- OK, let’s start.

We would like to hear from you about your experience with the Lea Mimba club. We are going to ask some questions about what it was like and what you thought about it.

*Ask everyone in the group to introduce themselves briefly, and then go around and answer this question: After participants have finished introducing themselves, proceed with asking the remaining questions:*

1. How did you hear about the Lea Mimba club?
2. Please tell me about your experience with the Lea Mimba club. Probes:

What did you like about the Lea Mimba Club?

- - Can you give me an example of what you liked?
  - What did you NOT like about the Lea Mimba Club?
  - Can you give me an example of what you did not like? How, if at all, did you benefit from the Lea Mimba club?

3. How easy was it for you to participate in the Lea Mimba club? Probes:

- Did you come for all of the sessions scheduled to-date?  Why or why not?
- How easy was it to remember your appointment and come on time? What might help you to more easily participate in the Lea Mimba club?

4. Please tell me about the nurse who ran the Lea Mimba club. Probes:

How well did he/she run the group sessions?

How would you describe your relationship with him/her? What did you like or not like about her/him?

5. Now I want to ask you about the other women in your group. What is your relationship like with them? Probes:

What did you think about having the ANC sessions with other women?

How, if at all, have your relationships with other women extended beyond the Lea Mimba sessions?

Now, I want to ask you some things that will help us to improve the Lea Mimba club.

6. What is the most important part of the Lea Mimba club to you?

7. Is there anything you would change about the Lea Mimba club? If so, what would you change?

1. What would you add?
2. What would you remove?

8.  How do you think we should let more women know about the Lea Mimba club? (*SKIP IF RUNNING OUT OF TIME)*

9.    Is there anything else that you would like us to know about the Lea Mimba club?

*Thank you for your time.*

**In-depth interview for group ANC client**

Sub County: _________________________________________________

Health Facility Name: ______________________________________

Date: ___________________________________________________

Data collector: __________________________________

Age: __________________________________________

Respondent parity: ________________________________________

Number of group ANC sessions attended: ________________________________

Gestational age (weeks): _______________________________________

Time started: ______________

Time ended: ______________

**Facilitator’s welcome, introduction and instructions to participants**
Welcome and thank you for volunteering to take part in this in-depth interview. My name is __________________, and I am conducting this interview on behalf of MSH to gather feedback on the Lea Mimba sessions. You have been asked to participate as your point of view is important. Participating is voluntary. I know you are busy and I appreciate your time. You will be compensated for your time at the end of this interview.

**Introduction:** This in-depth interview is designed to assess your thoughts about your experience in the Lea Mimba club sessions in which you participated. We would like to get your thoughts so that we can improve the Lea Mimba program. The interview will last for about 45 – 60 minutes. Your participation is entirely voluntary and you can leave the group discussion at any time. I will share with you an informed consent form for you to sign. We will be recording this session and taking notes so that we can capture everything you are saying. None of this data will have names or any personal information associated with it.

***SECURE INFORMED CONSENT***

Before we start, do you have any questions?

We would like to hear from you about your experience with the Lea Mimba club. We are going to ask some questions about what it was like and what you thought about it.

1. How did you hear about the Lea Mimba club?

Probes:

- Who told you about the Lea Mimba club the first time?
- What did they tell you?

1. Please tell me about your experience with the Lea Mimba club. Tafadhali, niambie mengi kuhusu kushiriki kwako kwenye klabu cha Lea Mimba

Probes

- What did you like about the Lea Mimba Club?
  - Can you give an example from one of your sessions?
  - Why?
- What did you NOT like about the Lea Mimba Club?
- Can you give an example from one of your sessions? Why?
- How did you benefit, if at all, from the Lea Mimba club?

3. Please tell me about the nurse who ran the Lea Mimba club. Probes

- - How well did he/she run the group sessions?
  - How would you describe your relationship with him/her?
  - What did you like or not like about her/him? Ni nini ulipenda au hukupenda kumhusu?

4. Now I want to ask you about the other women in your group. What is your relationship like with them? Probes:

- What did you think about having the ANC sessions with other women?
- How, if at all, have your relationships with other women extended beyond the Lea Mimba sessions?

5. What have you learned during the Lea Mimba club that has made a difference in your life? Probes:

*Keep probing for what else the participant might have learned. Ask “what else?” after everything they have said.*

- What is the most important part of the Lea Mimba club to you?
- How, if at all, has it made a difference in your life?
- How has it affected your health or your future newborn baby’s health?
- What challenges have you faced in trying to implement the lessons learned from the Lea Mimba club?
- What do you know about the timing and frequency of getting antenatal care during pregnancy?
  - When during your pregnancy did you begin antenatal care?
- At what time during pregnancy should a woman first seek antenatal care? How often does a woman need to seek antenatal care during a pregnancy?
- Do you think early ANC is important for the health of the mother and baby? Why or why not?
- Do you believe that going to ANC regularly (4 or more times) is important for the health of the mother and baby?

7. Have you talked about the Lea Mimba Clubs with your family and friends?

- - If NO, SKIP TO NEXT QUESTION.
  - If YES, ask questions below: How have they reacted to it?
    1. What did your husband say?
    2. What did your in-laws say?
    3. What did your friends say?

8. How easy was it for you to participate in the Lea Mimba club? Probes

- Did you come for all of the sessions scheduled to-date? Why or why not?
- How easy was it to remember your appointment and come on time?
- What were the challenges with coming to Lea Mimba group sessions?
- What might help you to more easily participate in the Lea Mimba club?

9. Now that you have been participating in a Lea Mimba club, if a pregnant friend or family member asked you about the Lea Mimba Club, what would you tell her about it?

10. Now I want to ask you some questions about comparing your Lea Mimba ANC experience with other ANC experiences.

IF NO, SKIP TO THE NEXT QUESTION..

IF YES, ASK:

How do you compare the Lea Mimba club to the usual ANC?

- What are the differences between the Lea Mimba club and the usual ANC?
  - Can you give me some examples?
- Which one do you prefer?
  - Why do you prefer it?

11.  Now, I want to ask you some things that will help us to improve the Lea Mimba club.

- - What is the most important part of the Lea Mimba club to you?
  - If you had to choose, what part of the Lea Mimba club would you remove?
  - What would you add or change that might make the Lea Mimba club better?
  - How do you think we should let more women know about the Lea Mimba club?

12. Would you choose to join the Lea Mimba club in your next pregnancy? Why or why not?

13. Would you recommend the Lea Mimba club to your sister, friend, family member or neighbour? Why or why not?

14.    Is there anything else that you would like us to know about the Lea Mimba club?

*Thank you for your time.*

**In-depth interview Guide for Health Facility Providers**

**Facilitator’s welcome, introduction and instructions to participants**

Welcome and thank you for volunteering to take part in this in-depth interview. My name is __________________ and I am conducting this interview on behalf of MSH to gather feedback on the Lea Mimba sessions. You have been asked to participate as your point of view is important. Participating is voluntary. I know you are busy and I appreciate your time. You will be compensated for your time at the end of this interview.

**Introduction:** This in-depth interview is designed to assess your thoughts about your experience in the Lea Mimba club sessions in which you participated. We would like to get your thoughts so that we can improve the Lea Mimba program. The interview will last for about 45 – 60 minutes. Your participation is entirely voluntary and you can leave the group discussion at any time. I will share with you an informed consent form for you to sign. We will be recording this session and taking notes so that we can capture everything you are saying. None of this data will have names or any personal information associated with it.

***SECURE INFORMED CONSENT***

Before we start, do you have any questions for me?

Sub County: _________________________________________________

Health facility name (if applicable): ______________________________________

Date: ___________________________________________________

Time interview started: _______

Time ended: _______

Interviewer/data collector: __________________________________

Type of respondent (tick box):

□ County health manager □ Health facility manager □ Health worker □ CHV

□ Other (specify :___________)

Respondent gender: □ Male □ Female

We would like to hear from you about your experience supporting implementation of the Lea Mimba club. We are going to ask some questions about what it was like and what you thought about it.

1. How have you been involved with the Lea Mimba club?

Probes:

What has been your experience with the Lea Mimba club?

*IF PARTICIPANTS HAVE* ***NOT*** *BEEN INVOLVED WITH THE LEA MIMBA CLUB, ABORT INTERVIEW.*

1. Please tell me what you know about the Lea Mimba   club

Probes:

- What is the purpose of the Lea Mimba club?
- How does the Lea Mimba club work?
- How is a typical Lea Mimba club session organized?
- What are the expected benefits?

1. What do you think about the Lea Mimba club?

Probes:

- What are the benefits of the Lea Mimba club?
- What are challenges with the Lea Mimba club?
- How do you compare the Lea Mimba club to the usual way of providing ANC?
  - What are the benefits of the Lea Mimba club when compared to individual ANC visits?
  - What are the inconveniences of the Lea Mimba club when compared to individual visits?

1. Now I want to ask you some questions about the relationships that formed during the Lea Mimba club.
2. Can you describe to me the relationships between participants of the Lea Mimba club?

Probes:

- How did women relate to each other?
- What interactions have you observed between women who attend the Lea Mimba club?

b. Can you describe to me your relationship with the participants of the Lea Mimba club?

Probes

- How was your relationship different from the usual way of providing ANC?
- How do you think the relationships with women participating in the Lea Mimba club affected how you provide ANC?

1. How did the implementation of the Lea Mimba club go in your health facility?

Probes:

- What was your plan to put it in place?
- What worked well?
- What was challenging?
- What did you do to address the challenges?

1. How would you describe the support you received to run the Lea Mimba club?

Probes:

- - What materials (e.g. picture cards, job aids, etc.)  did you find helpful?
  - What materials were not helpful?
  - Describe the support (e.g. training, supportive supervision, etc.) you received from the KPNA nurses.
  - What was helpful?
  - What additional support did you wish you had?

Now, I want to ask you some things that will help us to improve the Lea Mimba club.

1. What do you think is the most important part of the Lea Mimba club?

Probes:

- What elements of the club are essential?
- If you had to choose, what part of the Lea Mimba club would you remove?
- What aspects of the club are not essential or important?
- What would you add or change that might make the Lea Mimba club better for the health providers?
- What would you add or change that might make the Lea Mimba club better for the women who participate?
- What additional support or materials do you need to continue with the Lea Mimba club?
- If we wanted to expand to all health facilities in the district, what else might be needed?

1. Is there anything else that you would like us to know about the Lea Mimba club?

*Thank you for your time*

**Stakeholder In-depth interview Guide: Health Facility Manager**

**Facilitator’s welcome, introduction and instructions to participants**

Welcome and thank you for volunteering to take part in this in-depth interview. My name is __________________ and I am conducting this interview on behalf of MSH to gather feedback on the Lea Mimba sessions. You have been asked to participate as your point of view is important. Participating is voluntary. I know you are busy and I appreciate your time. You will be compensated for your time at the end of this interview.

**Introduction:** This in-depth interview is designed to assess your thoughts about your experience in the Lea Mimba club sessions in which you participated. We would like to get your thoughts so that we can improve the Lea Mimba program. The interview will last for about 45 – 60 minutes. Your participation is entirely voluntary and you can leave the group discussion at any time. I will share with you an informed consent form for you to sign. We will be recording this session and taking notes so that we can capture everything you are saying. None of this data will have names or any personal information associated with it.

***SECURE INFORMED CONSENT***

Before we start, do you have any questions for me?

Sub County: _________________________________________________

Health facility name (if applicable): ______________________________________

Date: ___________________________________________________

Time interview started: _______

Time ended: _______

Interviewer/data collector: __________________________________

Type of respondent (tick box):

□ County health manager □ Health facility manager □ Health worker □ CHV

□ Other (specify :___________)

Respondent gender: □ Male □ Female

We would like to hear from you about your experience supporting implementation of the Lea Mimba club. We are going to ask some questions about what it was like and what you thought about it.

1. How have you been involved with the Lea Mimba club?

Probes:

What has been your experience with the Lea Mimba club?

***IF PARTICIPANTS HAVE NOT BEEN INVOLVED WITH THE LEA MIMBA CLUB, ABORT INTERVIEW.***

2. Please tell me what you know about the Lea Mimba club

Probes:

- What is the purpose of the Lea Mimba club?
- How does the Lea Mimba club work?
- How is a typical Lea Mimba club session organized?
- How does it differ from the usual way of providing information and counseling for ANC clients?
- What are the expected benefits?

3. What do you think about the Lea Mimba club?

Probes:

- What are the benefits of the Lea Mimba club?
- What are challenges with the Lea Mimba club?
- How do you compare the Lea Mimba club to the usual way of providing ANC?
  - What are the benefits of the Lea Mimba club when compared to individual ANC visits?
  - What are the inconveniences of the Lea Mimba club when compared to individual visits?

4. How did implementation of the Lea Mimba club go in your health facility?

Probes:

- What was your plan to put it in place?
- What worked well?
- What was challenging?
- What did you do to address the challenges?

Now, I want to ask you some things that will help us to improve the Lea Mimba club.

5. What do you think is the most important part of the Lea Mimba club?

Probes:

- What elements of the club are essential?
- If you had to choose, what part of the Lea Mimba club would you remove?
- What aspects of the club are not essential or important?
- What would you add or change that might make the Lea Mimba club better for the health providers or the women who participate?
- What additional support or materials do you need to continue with the Lea Mimba club?
- If we wanted to expand to all health facilities in the district, what else might be needed?

6. Is there anything else that you would like us to know about the Lea Mimba club?

*Thank you for your time*
